# DPP4 Inhibitor Sitagliptin Enhances Lymphocyte Recruitment and Prolongs Survival in a Syngeneic Ovarian Cancer Mouse Model

**DOI:** 10.3390/cancers13030487

**Published:** 2021-01-27

**Authors:** Amy L. Wilson, Laura R. Moffitt, Kirsty L. Wilson, Maree Bilandzic, Mark D. Wright, Mark D. Gorrell, Martin K. Oehler, Magdalena Plebanski, Andrew N. Stephens

**Affiliations:** 1 Centre for Cancer Research, Hudson Institute of Medical Research, Clayton 3168, Australia; amy.wilson@hudson.org.au (A.L.W.); laura.moffitt@monash.edu (L.R.M.); maree.bilandzic@hudson.org.au (M.B.); 2 Department of Molecular and Translational Sciences, Monash Health, Clayton 3168, Australia; 3 Department of Immunology and Pathology, Monash University, Clayton 3800, Australia; mark.wright@monash.edu; 4 School of Health and Biomedical Sciences, RMIT University, Bundoora 3083, Australia; kirsty.wilson2@rmit.edu.au; 5 Centenary Institute, Faculty of Medicine and Health, University of Sydney, Camperdown 2006, Australia; m.gorrell@centenary.org.au; 6 Department of Gynaecological Oncology, Royal Adelaide Hospital, Adelaide 5000, Australia; martin.oehler@adelaide.edu.au

**Keywords:** sitagliptin, ovarian cancer, DPP4, ID8, syngeneic, immune, T-cell

## Abstract

**Simple Summary:**

The role of immunity in the development and progression of epithelial ovarian cancer (EOC) is well established. Poor T-cell infiltration is associated with mortality in EOC patients, and recent evidence has suggested that the enzyme DPP4 plays a role in this process. The aim of our study was to evaluate the potential of the clinically-approved DPP4-inhibitor sitagliptin to improve immune responses in mice with EOC. We showed that sitagliptin improved CD8+ T-cell responses in an EOC mouse model, consequently reducing metastatic burden and prolonging survival. These data provide a rationale for the use of DPP4-inhibitors as a second-line treatment for EOC.

**Abstract:**

Immunity plays a key role in epithelial ovarian cancer (EOC) progression with a well-documented correlation between patient survival and high intratumoral CD8+ to T regulatory cell (Treg) ratios. We previously identified dysregulated DPP4 activity in EOCs as a potentially immune-disruptive influence contributing to a reduction in CXCR3-mediated T-cell infiltration in solid tumours. We therefore hypothesized that inhibition of DPP4 activity by sitagliptin, an FDA-approved inhibitor, would improve T-cell infiltration and function in a syngeneic ID8 mouse model of EOC. Daily oral sitagliptin at 50 mg/kg was provided to mice with established primary EOCs. Sitagliptin treatment decreased metastatic tumour burden and significantly increased overall survival and was associated with significant changes to the immune landscape. Sitagliptin increased overall CXCR3-mediated CD8+ T-cell trafficking to the tumour and enhanced the activation and proliferation of CD8+ T-cells in tumour tissue and the peritoneal cavity. Substantial reductions in suppressive cytokines, including CCL2, CCL17, CCL22 and IL-10, were also noted and were associated with reduced CD4+ CD25+ Foxp3+ Treg recruitment in the tumour. Combination therapy with paclitaxel, however, typical of standard-of-care for patients in palliative care, abolished CXCR3-specific T-cell recruitment stimulated by sitagliptin. Our data suggest that sitagliptin may be suitable as an adjunct therapy for patients between chemotherapy cycles as a novel approach to enhance immunity, optimise T-cell-mediated function and improve overall survival.

## 1. Introduction

Epithelial ovarian cancer (EOC) is the most lethal gynaecological malignancy and the sixth-leading cause of cancer-related deaths in females [1]. Standard treatments for ovarian cancer involve highly invasive surgery and concurrent platinum (carboplatin and cisplatin) and taxane (paclitaxel) chemotherapies, to which the majority of patients initially respond favourably [2]. However, disease recurrence and acquired chemoresistance is almost universal, limiting therapeutic options and accounting for the disproportionally high mortality rate of EOC [3]. Novel therapeutic strategies for EOC include endocrine therapy, which has thus far shown reasonable therapeutic responses [4] and immunotherapies. However, their further development for the treatment of EOC is urgently required to improve survival rates.

Whilst substantial evidence supports an important and complex role for immunity in the development and progression of EOC [5], immunotherapy currently lacks efficacy for EOC due to immune evasion and subsequent poor lymphocyte infiltration and dysfunction [6]. Tumour-infiltrating lymphocytes (TILs) including cytotoxic CD8+ T effector (Teff) cells are recruited to the ovarian tumour by expression of chemokines such as CXCL10 in a CXCR3-mediated manner and facilitate antitumour immune responses by producing interferon (IFN)-γ and by releasing the content of cytolytic granules for the direct elimination of cancer cells [7]. By contrast, T regulatory cells (Tregs) impair antitumour immunity by secreting cytokines such as TGF-ß and IL-10 and by inhibiting the production and activity of proinflammatory cytokines [8]. Accordingly, a high ratio of Treg to Teff cells is a strong predictor of poor prognosis in ovarian cancer patients [9]. Whilst active immunosuppression within the ovarian tumour microenvironment thus promotes tumour progression, its targeted disruption holds significant potential to improve immunotherapeutic efficacy for EOC treatment.

Dysregulation of the type II transmembrane glycoprotein dipeptidyl peptidase 4 (DPP4) has been implicated in several cancer types including urothelial carcinoma [10], papillary thyroid carcinoma [11], metastatic prostate cancer [12] and epithelial ovarian cancer [13]. DPP4 can enzymatically truncate proteins containing either L-proline or L-alanine at the penultimate position, and for this reason plays a major role in glucose metabolism where it regulates glucagon-like peptide-1 (GLP1) and gastric inhibitory protein (GIP) [14]. Accordingly, several DPP4 inhibitors such as sitagliptin are approved for the management of type 2 diabetes, as DPP4 inhibition in this context prolongs the half-life of GLP1 and GIP, increasing endogenous insulin secretion [15]. Other DPP4 substrates include neuropeptide Y, substance P and chemokines such as CXCL9, CXCL10, CXCL11 and CXCL12 [16,17,18]. In particular, DPP4 plays an important role in the regulation of CXCL10 function and associated T cell recruitment in patients with hepatitis C virus (HCV) [19]. Previous work has demonstrated preserved bioactive CXCL10 in healthy individuals receiving the clinically approved DPP4 inhibitor sitagliptin, thereby establishing its role in CXCL10 post-translational modification [20]. Furthermore, mice bearing melanoma and colorectal tumours treated with sitagliptin showed improved naturally occurring antitumour immune responses via preservation of bioactive CXCL10, a chemokine that is essential for lymphocyte chemotaxis [21].

DPP4 overexpression has been established in EOC [13,18]. However the use of DPP4 inhibitors as a novel immunotherapy for this disease has not yet been shown. In the current study, we sought to determine whether DPP4 inhibition using sitagliptin enhances lymphocyte tumour infiltration in a syngeneic mouse model of EOC [22] to abrogate tumour-mediated immune suppression and promote antitumour immunity. In the current study, we demonstrated delayed tumour metastasis and increased survival with associated increases in CD8+ T-cell activation, proliferation and CXCR3-mediated T-cell recruitment to ovarian tumour tissue. Overall, our findings provide proof of principle evidence for the repurposing of DPP4 inhibitors as a novel ovarian cancer immunotherapy.

## 2. Methods

### 2.1. Cell Culture

The ID8 mouse epithelial ovarian cancer cell line (a gift from Dr. Kathy Roby, Kansas University Medical Center) was maintained in DMEM (Gibco, Palo Alto, CA, USA) containing 4% fetal bovine serum (FBS), 1% penicillin/streptomycin (PS) and 1x insulin-transferrin-selenium (ITS). *pROSA*-iRFP720 ID8 cells were generated and maintained as previously described [22].

### 2.2. Animal Experiments

Female 8-week-old C57BL/6 mice were obtained from Monash Animal Services (Melbourne, VIC, Australia) and housed in a specific-pathogen-free (SPF) facility. All animal protocols were approved by the Alfred Medical Research and Education Precinct (AMREP) animal ethics committee, Melbourne, Australia (approval #E/1682/2016/M). Treatment and care of the animals were in accordance with institutional guidelines and with the Australian code for the care and use of animals for scientific purposes.

Mice were supplemented with an SF-AIN-93M rodent diet to reduce intrinsic autofluorescence. Mice (*n* = 5/group) were divided into four treatment groups (untreated, sitagliptin, paclitaxel, sitagliptin-paclitaxel combination) and three time points (early-stage disease, metastatic disease, survival) (Supplementary Figure S1). For treatments, mice were given food containing 50 mg/kg/day equivalent sitagliptin (Januvia^®^, Merck, Kenilworth, NJ, USA) (plus 25 mg/kg and 100 mg/kg for optimisation studies) from week two post-tumour implantation. For combination treatment studies, mice receiving sitagliptin were also administered two doses of intraperitoneal paclitaxel (15 mg/kg) (Pfizer Inc., New York, NY, USA) at week three and four post-tumour implantation. Mice were monitored weekly for weight and circumference, and humane endpoints were determined by a body abdominal circumference of > 100 mm and general wellbeing (lack of responsiveness, hunched posture, piloerection, eyes squinted). Once endpoint was reached, mice were humanely sacrificed by CO_2_ asphyxiation. Blood was processed for serum for cytokine analysis, and red blood cells were lysed for leukocyte analysis by flow cytometry. Brachial and inguinal lymph nodes, spleen and peritoneal washes were harvested for flow cytometry, and ovarian tumour and metastatic tumour tissue were either formalin-fixed and embedded in paraffin for immunofluorescence or preserved in optical cutting temperature (O.C.T.) compound for DPP4 in situ enzyme activity assays.

### 2.3. Intrabursal Implantation of ID8 pROSA-iRFP720 Tumours

Tumours were established by ovarian intrabursal (IB) implantation of *pROSA*-iRFP720 ID8 cells as described previously [22,23]. Mice were anaesthetised in an induction chamber using 3% isofluorane in 1L/min oxygen and then maintained at 2% isofluorane in 0.3 L/min oxygen using a rodent facemask. An incision was made at the mid-dorsal region of the skin and the peritoneal membrane was excised at the latero-dorsal point above the location of the right ovary. The ovarian fat pad was externalised and stabilised with a serrefine clamp. 1 × 10^6^ *pROSA*-iRFP720 ID8 cells were loaded into a Hamilton microliter syringe (Sigma-Aldrich, St. Louis, MO, USA) and injected underneath the ovarian bursa using a dissecting microscope for guidance. The skin was closed using Michel suture clips. Mice were monitored for recovery, and suture clips were removed seven days postsurgery.

### 2.4. In Vivo Fluorescence Imaging

In vivo near-infrared fluorescence imaging was performed weekly using an IVIS Lumina III in Vivo Imaging System as described previously [22]. Briefly, *pROSA*-iRFP720 ID8 tumour-bearing mice were anesthetised using isofluorane in oxygen and were imaged at field of view (FOV) C with brightfield and X-ray set on auto-exposure. iRFP720 fluorescence was detected using the spectral unmixing iRFP filter set (excitation 620 nm, 640 nm, 660 nm, 680 nm; emission 710 nm), at an exposure time of 5 s. Spectral unmixing of iRFP720 signal from intrinsic autofluorescence was performed by subtracting 680_ex_/710_em_ fluorescence values from the other channels using an established custom spectral unmixing algorithm. All quantitative fluorescence measurements and analyses were performed using the Living Image software (v 4.5.1, PerkinElmer, Waltham, MA, USA).

### 2.5. Measurement of Plasma DPP4 Enzyme Activity

DPP4 enzyme activity in mouse serum was measured using H-Gly-Pro-pNA (Sigma-Aldrich, MO, USA) as previously described [18] Human recombinant DPP4 (Abcam, Cambridge, England) was used as a positive control for DPP4 enzyme activity, and recombinant DPP4 treated with 1µM sitagliptin (Januvia^®^) was used as a control for enzyme inhibition. A standard curve was constructed using pNA (Sigma-Aldrich, MO, USA) ranging from 1.56 mM–50 mM. All measurements were carried out in duplicate. Absorbance at 405 nm and 570 nm was measured every 5 min for 3 h at 37 °C using a Cytation 3 MultiMode Plate Imager (BioTek^®^ Instruments Inc., Winooski, VT, USA). Absorbance values at 405nm were subtracted from readings at 570 nm to account for potential optical interference. Standard curves were constructed using Gen5.0 software v2.05 (BioTek Instruments Inc., Winooski, VT, USA).

### 2.6. In Situ Staining of DPP4 Activity

For evaluation of in situ DPP4 activity, 10 µm frozen cryosections from ovarian tumours were air dried and incubated with 1.5 mM glycyl-prolyl-4-methoxy-β-naphthylamide and 2.5 mM Fast Blue BB Salt in assay buffer (50 mM Tris, 150 mM NaCl, 0.05% Tween 20 at pH 7.6) for 30 min at 37 °C [24]. Sections were washed with assay buffer, nuclei were stained using Harris haematoxylin (Sigma-Aldrich, MO, USA) and slides were mounted with water. Slides were immediately imaged with a Nikon Eclipse 50i microscope (Nikon, Tokyo, Japan) using a 10× and 20× objective, and captured with a Nikon DS-Fi1 camera (Nikon, Tokyo, Japan). Ovarian tumour and liver sections treated with 1 µM sitagliptin (Januvia^®^) served as negative controls, and liver sections served as positive controls. Images were analysed using ImageJ v1.0 (National Institute of Health, Rockville, MD, USA) for percentage of area and integrated density.

### 2.7. Flow Cytometry

Leukocytes from lymph nodes, blood, spleen and peritoneal cavity wash were collected and isolated as described [25], and analysed by flow cytometry. Antimouse CD16/CD32 antibody (1:50, BD Biosciences, clone #2.4G2) was used to block nonspecific binding. Zombie Aqua™ fixable viability dye (BioLegend, San Diego, CA, USA) was used to distinguish live and dead cells. The following fluorochrome-conjugated antibodies were used: CD3e-AF700 (1:200, BD Biosciences, clone #500A2), CD4-BUV395 (1:100, BD Biosciences, clone #RM4-5), CD8a-PerCP (1:100, BioLegend, clone #53-6.7), CD25-PE-CF594 (1:100, BD Biosciences, #PC-61), FoxP3-APC (1:50, eBioscience, clone #FJK-16S), CD11b-APC (1:200, BioLegend, clone #MI/71), CD11c-BV421 (1:50, BD Biosciences, clone #HL3), GR1-PerCP Cy5.5 (1:200, BD Biosciences, clone #RB6-8C5), F4/80-BV711 (1:100, BioLegend, clone #BM8), CD69-APC/Cy7 (1:100, BD Biosciences, clone #HI-2F3), Ki67-BV786 (1:100, BD Biosciences, clone #B56), CCR4-BV421 (1:100, Biolegend, clone #2G12), and CXCR3-BV605 (1:100, BioLegend, clone #CXCR3-173). Single stained beads were used to set compensation controls, and fluorescence minus one (FMO) controls were used to define population gates (Supplementary Figure S2). FMO1: CD3, CD4, CD8; FMO2: CD3, CD4, CD8, CD25, FoxP3; FMO3: CD11c, F4/80, GR1. Data were acquired using a BD LSRFortessa X-20 (BD Biosciences, Franklin Lakes, NJ, USA), and was analysed using FlowJo software v10.5.0 (LCC, Ashland, OR, USA).

### 2.8. Tissue Immunofluorescent Staining

Immunofluorescent staining was performed on 4µm formalin-fixed, paraffin-embedded tumour tissue microarrays (TMAs), constructed by Monash Health, Clayton, Australia. Following deparaffinisation, antigen retrieval was performed by boiling slides in sodium citrate buffer, and permeabilisation was performed using 0.25% *v/v* Triton X-100. Nonspecific antibody binding was blocked with 6% normal serum in TBS/0.25% Triton X-100, and primary antibodies were applied and incubated overnight at 4 °C. Fluorochrome-conjugated antibodies used were: CD3-AlexaFluor^®^647 (30 μg/mL, BD Biosciences, clone #17A2), CD4-eFluor660 (10 μg/mL, eBioscience, clone #4SM95), CD8a-eFluor615 (20 μg/mL, eBioscience, clone #53-6-7), CD69-AlexaFluor^®^488 (30 μg/mL, BioLegend, clone #H1-2F3) and CXCR3 (5 μg/mL, GeneTex, Irvine, CA, USA) plus goat antirabbit IgG H + L-AlexaFluor^®^488 secondary antibody (Abcam, Cambridge, UK). Sudan Black B (Sigma-Aldrich, MO, USA) (0.3% in 70% ethanol) was used to reduce intrinsic autofluorescence. Nuclei were stained with DAPI (ThermoFisher Scientific, Walsham, MA, USA) prior to mounting with DPX (Sigma-Aldrich, MO, USA). Fluorescence images were captured using the VS120 Virtual Slide Microscope (Olympus Corporation, Shinjuku City, Tokyo, Japan) by the Monash Health Translation Precinct (MHTP) Histology Facility, Clayton, Australia. Images were processed using the Olyvia software v2.9.1 (Olympus Corporation, Japan) and analysed using the ImageJ v1.0 software (National Institute of Health, Bethesda, MD, USA) for colocalisation of CD4+CXCR3+, CD8+CXCR3+, and CD3+CD8+CD69+ (percentage of area).

### 2.9. Chemokine Luminex Assay

Quantitation of 31 cytokines in mouse serum was determined using a Bio-Plex Pro™ Mouse Chemokine Panel 31-Plex (Bio-Rad, Hercules, CA, USA) as described [26]. The assay was performed using mouse serum diluted at 1:5 in sample diluent, data acquisition was performed using the Bio-Plex 200 reader (Bio-Rad, CA, USA) and was analysed using a two-group comparison of fold-change on the Qlucore Omics Explorer v3.5 software (Qlucore AB, Lund, Sweden).

### 2.10. Statistical Analysis

All statistical analyses were performed using GraphPad Prism v8.0.2 (GraphPad Software Inc., San Diego, CA, USA) unless otherwise stated. A two-way ANOVA was used to analyse statistical significance between groups for DPP4 activity and analysis of small intestine-associated tumour nodules. A two-way ANOVA and Sidak multiple comparisons test were used to analyse statistical significance between groups for flow cytometry immune populations. A log-rank (Mantel-Cox) test was used to analyse statistical significance between groups for the Kaplan-Meier survival curve, with approximate 95% confidence intervals calculated for the sample medians. A two-group comparison was used to analyse statistical significance between cytokines using the Qlucore Omics Explorer v3.5 software (Qlucore AB, Lund, Sweden). Data are presented as mean ± SD unless otherwise stated, and *p* < 0.05 was considered statistically significant.

## 3. Results

### 3.1. Sitagliptin Alters DPP4 Activity and Localisation in Tumour Tissue

**Figure 1:**
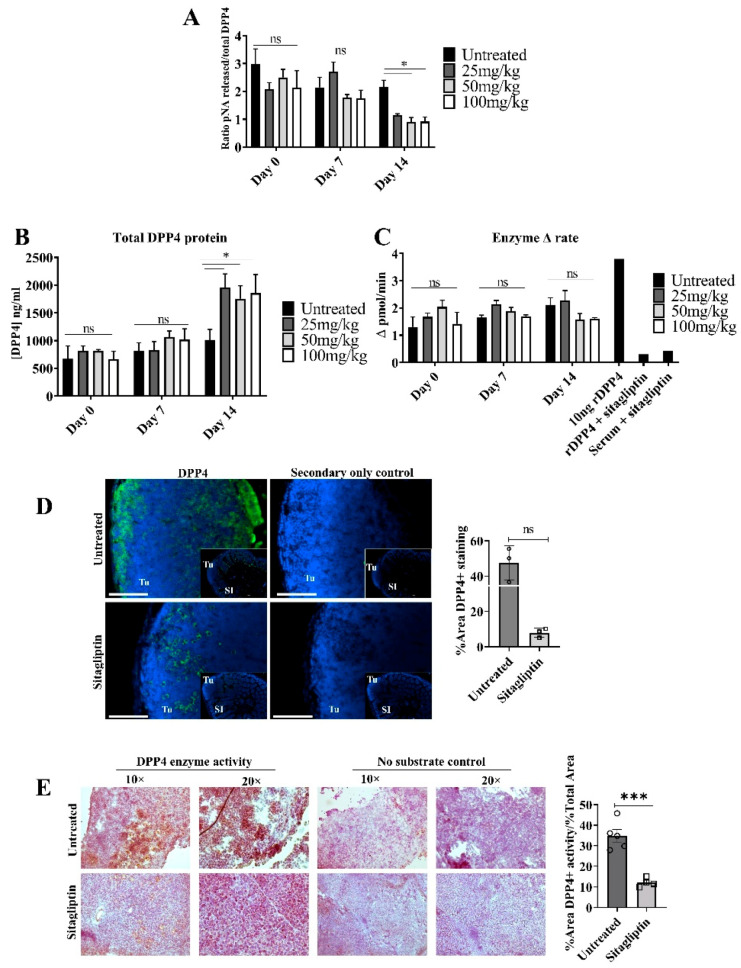
Circulating soluble and tumour-specific DPP4 expression and enzyme activity in mice treated with sitagliptin. ID8 iRFP+ epithelial ovarian tumours were intrabursally implanted into C57BL/6 mice and were allowed to develop for two weeks. Mice received daily oral sitagliptin treatment (25, 50 and 100 mg/kg) and circulating DPP4 protein expression and enzyme activity was measured. (**A**) Specific DPP4 activity in serum from mice treated with 25, 50, 100 mg/kg/day of sitagliptin at days 0, 7 and 14 following treatment. Specific DPP4 activity was determined by calculating the ratio of DPP4 enzyme activity (pmol/mL) to total protein concentration (ng/mL). (**B**) DPP4 protein concentration (ng/mL) was determined using a mouse anti-DPP4 ELISA kit. (**C**) Soluble DPP4 enzyme activity was determined by measuring the amount of p-nitronaniline (pNA) cleaved by DPP4 from the substrate H-Gly-Pro-pNA. (**D**) Representative fluorescent images of small intestine-associated metastases from mice bearing ID8 pROSA-iRFP720 tumours, treated with or without 50 mg/kg/day sitagliptin. DPP4 staining is shown in green and nuclei are stained with DAPI (blue). SI = small intestine; Tu = tumour. Scale bar represents 100 μm. Images were acquired using the Cytation 3 imaging Multi-Mode Reader (BioTek, VT, USA) and were processed using the Gen5™ software v2.05 (BioTek, VT, USA). Bar graph shows the percentage of DPP4-positive staining in metastatic small intestine-associated metastases (*n* = 3). (**E**) Tumour in situ DPP4 activity in mice bearing ID8 *pROSA*-iRFP720 tumours treated with 50mg/kg/day sitagliptin. In situ DPP4 activity was detected using Gly-Pro 4-methoxy-β-naphthylamide as a substrate, and nuclei were stained with haematoxylin. The bar graph indicates quantitated in situ DPP4 activity (*n* = 5). Data are presented as mean ± SD. ns = no significance; * = *p* < 0.05; *** = *p* < 0.001.

To establish the optimal concentration of sitagliptin required to inhibit systemic and tumour-localised DPP4 activity, mice bearing ovarian tumours were treated with 25, 50 or 100 mg/kg/day (oral, in food) sitagliptin for two weeks. DPP4 protein expression and activity were measured in serum and in ovarian tumour tissue. 50 mg/kg/day was the minimum concentration required to significantly reduce DPP4 specific activity in circulation two weeks following treatment initiation, with an approximate 58% reduction (Figure 1A). Total DPP4 inhibition was not reached at any of the concentrations examined. Interestingly, we observed that total soluble DPP4 (sDPP4) protein increased following sitagliptin treatment (Figure 1B) with no accompanying change in enzyme activity (Figure 1C). This supports previous data demonstrating the upregulation of sDPP4 levels following enzyme inhibition in diabetic mice [27].

We also evaluated the effect of sitagliptin on DPP4 expression and activity in tumour tissues in situ. Unlike sDPP4 in circulation, there was a decreasing trend in DPP4 abundance in tumour tissue two weeks following the commencement of sitagliptin treatment (Figure 1D). Unexpectedly, DPP4 localization was also altered; in untreated tumours, DPP4 staining was largely associated with the periphery of tumour mass, whilst in sitagliptin-treated mice, peripheral localization was absent and DPP4 staining was localised inside the tumour mass (Figure 1D). In situ DPP4 enzyme activity was also substantially reduced in sitagliptin-treated mice, in agreement with reduced local abundance (Figure 1E).

### 3.2. Sitagliptin Reduces Overall Tumour Burden and Prolongs Survival

**Figure 2:**
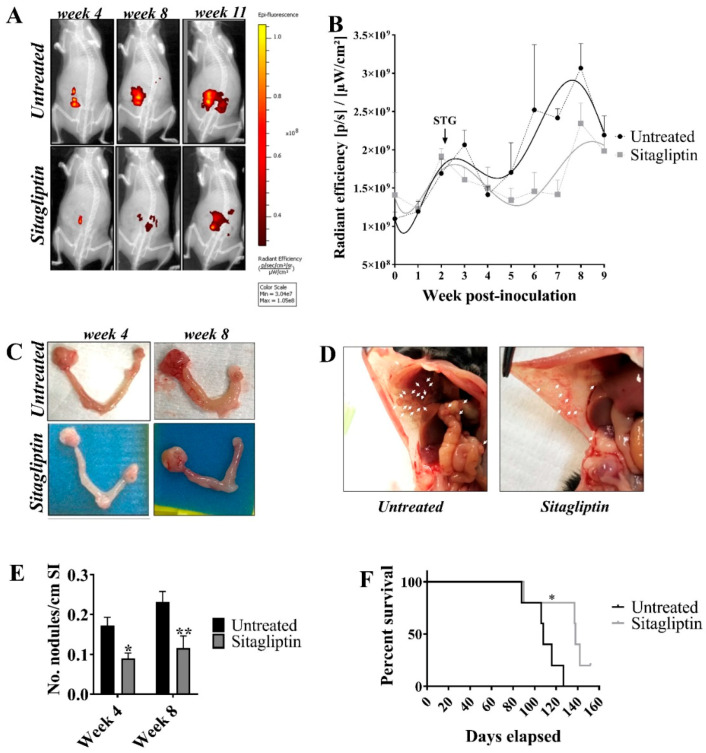
Tumour burden and metastatic spread in ID8 pROSA-iRFP720 tumour-bearing mice following sitagliptin treatment. (**A**) ID8 *pROSA*-iRFP720 cells were implanted into the ovarian bursa and iRFP720 fluorescence was measured using the IVIS Lumina III In Vivo Imaging System (Perkin Elmer, Waltham, MA, USA). Mice were imaged at FOV C (*n* = 5–15/group) and iRFP720 fluorescence was detected using the iRFP filter set at an exposure time of 5 s. iRFP signal was isolated using a custom spectral unmixing algorithm. Representative images of iRFP fluorescence are shown. (**B**) Quantitative region of interest (ROI) analysis of iRFP total radiant efficiency [p/s]/[µW/cm^2^] over time. (**C**) Representative images of ovaries from untreated mice or mice receiving sitagliptin at weeks 4 and 8 post tumour inoculation. ID8 *pROSA*-iRFP720 tumours are shown on the right and nontumour bearing ovaries are shown on the left. (**D**) The proportion of macroscopic tumour nodules on the small intestine/length (cm). (**E**) Representative images of tumour nodules on the peritoneal wall from untreated mice and mice receiving sitagliptin treatment. (**F**) The Kaplan-Meier curve and log-rank test of overall survival analysis for ID8 *pROSA*-iRFP720 tumour-bearing mice. Data are presented as mean ± SD (upper SD for iRFP720 fluorescence). * = *p* < 0.05; ** = *p* < 0.01.

Using our validated ID8 iRFP720 syngeneic ovarian cancer model [22], we evaluated the effect of sitagliptin treatment on ovarian cancer progression and overall survival. Mice with primary ID8 *pROSA*-iRFP720 ovarian tumours were treated with 50 mg/kg/day of oral sitagliptin, commencing two weeks post-implant, until experimental endpoint. Whilst sitagliptin treatment did not alter primary tumour volume (Figure 2A–C), there was a significant reduction in visible metastatic deposits compared to untreated controls (Figure 2D,E). Consistent with progression in this model, untreated mice developed extensive, disseminated metastatic deposits throughout the peritoneum, macroscopic tumour deposits on the liver, signs of anaemia (pale liver and extremities) and an accumulation of ascites fluid at eight weeks post-implant. By contrast, sitagliptin-treated mice had significantly fewer visible metastatic nodules and did not display signs of anaemia (Figure 2D,E). Overall, reducing DPP4 activity with sitagliptin reduced metastatic tumour burden in vivo.

The impact of daily sitagliptin treatment on overall survival was also assessed. Mice received daily oral sitagliptin until they became moribund due to ascites fluid accumulation. Sitagliptin treated mice had a median survival of 138 days (95% CI = 90–152), compared to 108 days (95% CI = 88–127) in the untreated controls (Figure 2F). The experiment overall was terminated at day 152, with a single sitagliptin-treated animal euthanised before reaching humane endpoint. Thus, daily oral sitagliptin significantly improved overall survival of mice (*n* = 5/group) with ID8 *pROSA*-iRFP720 epithelial tumours (*p* = 0.0269).

### 3.3. Sitagliptin Alters the Immune Landscape During Early-Stage Disease

**Figure 3:**
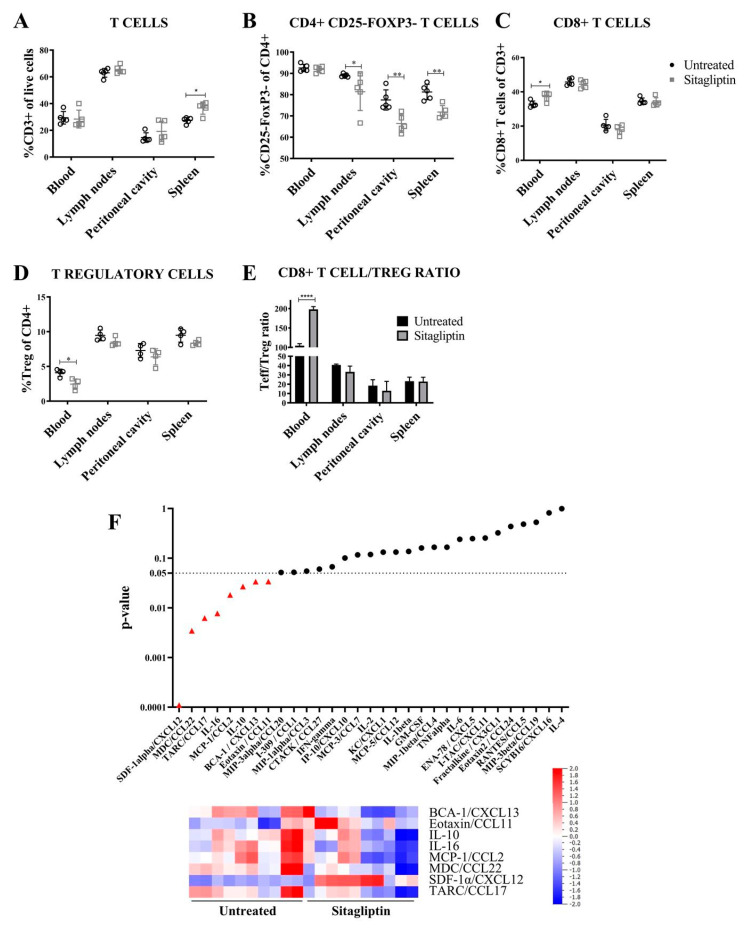
Gross lymphocyte populations and circulating cytokines in mice treated with sitagliptin. Leukocytes were isolated from the blood, lymph nodes, peritoneal cavity and spleen of ID8 *pROSA*-iRFP720-bearing mice at week four post-inoculation and examined using a BD LSRFortessa X-20 (BD Biosciences). (**A**) Percentage of CD3+ T cells of live cells. (**B**) Percentage of CD4+ T cells (CD3+CD4+CD25-FoxP3-) of CD4+ cells. (**C**) Percentage of CD8+ T cells (CD3+CD8+) of CD3+ cells. (**D**) Percentage of T regulatory cells (CD3+CD4+CD25+FoxP3+) of CD4+ cells. (**E**) CD8+ T cell/T regulatory cell ratio. Data are presented as mean ± SD, *n* = 5. * = *p* < 0.05; ** = *p* < 0.01; **** = *p* < 0.0001. (**F**) Cytokines significantly altered in the serum of mice receiving sitagliptin examined at week four post tumour inoculation. The heat map represents cytokines with significant changes between the untreated and sitagliptin-treated mice (individual mice shown). Blue indicates the lowest value while red indicates the highest value. Data analysed using Qlucore Omics Explorer v3.5 (Qlucore AB, Lund, Sweden).

To investigate the effect of sitagliptin on lymphocyte populations during early stage tumour dissemination [22], lymphocyte populations in the spleen, lymph nodes, blood and peritoneal tumour environment were assessed four weeks post-implant (two weeks post-sitagliptin initiation). At this stage, sitagliptin induced a significant increase splenic in CD3+ T cells, while CD3+ T cells remained unchanged in other tissues examined (Figure 3A). T cell lineage was altered, however, with decreased proportions of CD4+CD25-FoxP3- T cells observed in the lymph nodes, peritoneal cavity and spleen (Figure 3B). The %CD8+ T cell in blood also increased (Figure 3C), with a reciprocal decrease in %Tregs (Figure 3D). As a consequence, the overall CD8+ T cell/Treg ratio in circulation was increased (Figure 3E), consistent with clinical data associating these changes with a favourable prognosis [28]. A concomitant decrease in myeloid-derived suppressor cell (MDSC) proportions was also observed (Supplementary Figure S3A).

Cytokine analysis also revealed treatment-specific changes in serum, largely involving cytokines with immunosuppressive properties (Figure 3F). CCR4 ligands CCL22 and CCL17, each involved in Treg recruitment into the tumour microenvironment (TME) [29], were comparatively decreased in mice treated with sitagliptin (Figure 3F) and were consistent with the observed decrease in Treg abundance (Figure 3D). Decreased abundance of IL-10, IL-16 and CCL2 (Figure 3F), each with immune suppressive properties [30,31], were also noted. By contrast, the DPP4 substrate CXCL12 increased in mice receiving sitagliptin treatment (Figure 3F), consistent with several in vivo studies [32,33]. Abundance increases in the B cell and eosinophil chemotactic ligands CXCL13 and CCL11, respectively, (Figure 3F) were also observed. Taken together, these data show that sitagliptin (i) increased CD8+ T cell/Treg ratios and (ii) reduced the expression of immunosuppressive cytokines, thereby altering the entire circulating immune landscape during the early dissemination stages of ovarian cancer.

### 3.4. Sitagliptin Induces Activation and Proliferation of Peripheral and Intra-Tumoral CD8+ T Cells

**Figure 4:**
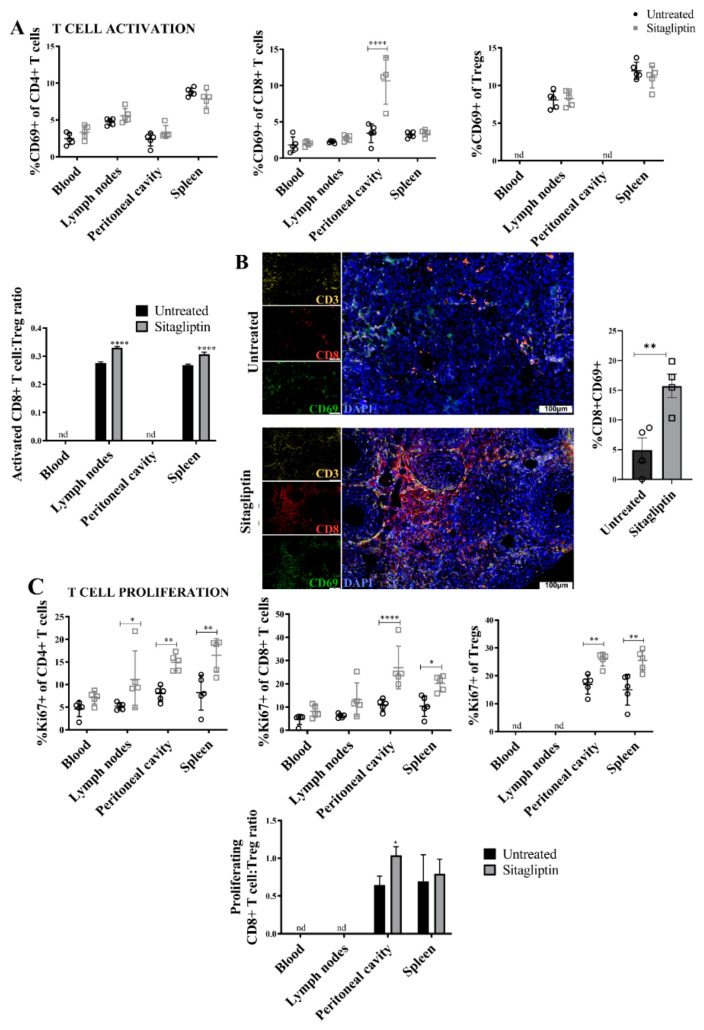
Lymphocyte activation and proliferation in ID8 tumour-bearing mice treated with sitagliptin. Leukocytes were isolated from the blood, lymph nodes, peritoneal cavity and spleen of ID8 *pROSA*-iRFP720-bearing mice at week four post-inoculation and examined using a BD LSRFortessa X-20 (BD Biosciences). (**A**) Percentage of activated (CD69+) CD4+ T cells, CD8+ T cells, and T regulatory cells of the corresponding parent population, and ratio of CD69+ CD8+ T cells to CD69+ Tregs in each of the compartments examined. (**B**) Representative images of ovarian tumour sections stained with CD3 (yellow), CD8 (red) and CD69 (green) at week four post tumour inoculation. Nuclei were stained with DAPI (blue). Bar graph shows the percentage area of ovarian tumour sections stained with CD8+CD69+. Images were acquired using the VS120 Virtual Slide Microscope (Olympus Corporation, Japan) and processed using the Olyvia software v2.9.1 (Olympus Corporation, Japan). Data were analysed by calculating the percentage area of CD8+CD69+ colocalisation of total tissue area using a consistent binary threshold in ImageJ v1.0 (National Institute of Health, MD, USA). (**C**) Percentage of proliferating (Ki67+) CD4+ T cells, CD8+ T cells, and T regulatory cells of the corresponding parent population, and ratio of Ki67+ CD8+ T cells to Ki67+ Tregs in each of the compartments examined. Flow cytometry data are presented as mean ± SD, *n* = 5. * = *p* < 0.05; ** = *p* < 0.01; **** = *p* < 0.0001.

Functional CD8+ T-cell status was next assessed using flow cytometry to detect the activation and proliferation markers CD69 and Ki67, respectively. There was a significant increase in the percentage of CD69+ peritoneal CD8+ T-cells in sitagliptin treated mice (Figure 4A), in addition to an increased CD69+CD8+ T cell/Treg ratio in the lymphoid organs (Figure 4A). Blood and peritoneal ratios were not examined due to insufficient CD69+ Treg numbers. Immunofluorescence staining of tumour tissue confirmed that CD69+CD8+ T cells were increased in abundance in situ and localised to the primary tumour at this early stage (Figure 4B). Sitagliptin also altered proliferation, with a substantial increase in Ki67+CD4+ and Ki67+CD8+ T cells (both Teff and Tregs) in the spleen and peritoneal cavity. Sitagliptin also increased the %Ki67+CD4+ T cells in lymph nodes (Figure 4C). As a consequence, the ratio of proliferating Ki67+CD8+ T cell/Tregs in the peritoneal microenvironment increased (Figure 4C). Together, these data indicate that sitagliptin improved lymphocyte activation, proliferation and in situ accumulation in tumour tissues. These changes resulted in a beneficial increase in Teff/Treg ratios and are likely to positively influence anti-tumour immunity.

### 3.5. Enhanced CXCR3-Mediated Recruitment of CD8+ T Cells Is Abrogated by Paclitaxel

Previous work demonstrated that CXCL10, an established DPP4 substrate, mediates T-cell migration and recruitment to tumour tissue in vivo [34,35]. Moreover, reduction of DPP4 activity using sitagliptin can protect the biological activity of CXCL10 to enhance T-cell recruitment into tumours and improve overall survival [21]. We therefore examined CXCR3 expression on T cells as surrogate marker of CXCL10-mediated trafficking, as well as CCR4, a key mediator of Th2 and Treg recruitment [36].

**Figure 5:**
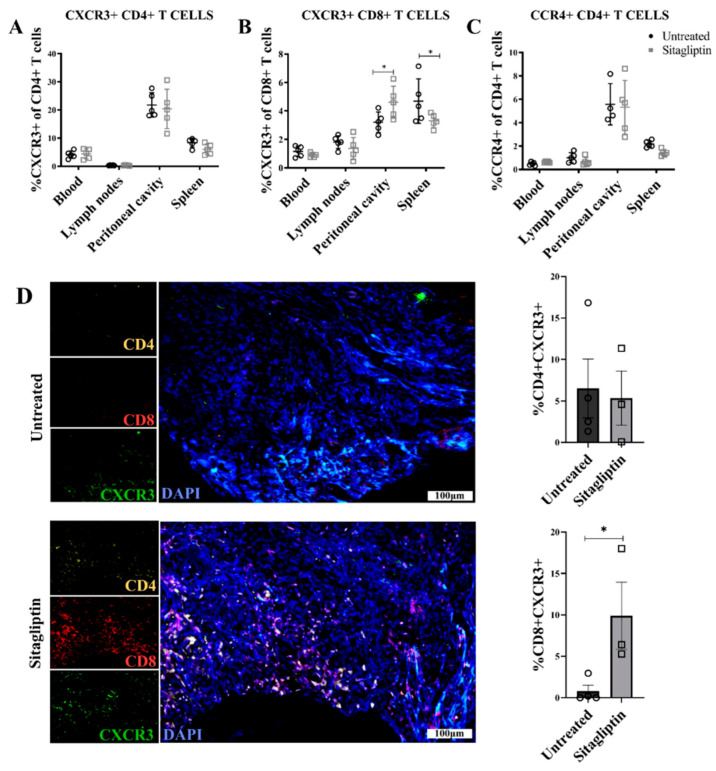
Chemokine receptor expression in ID8 tumour-bearing mice treated with sitagliptin. Leukocytes were isolated from the blood, lymph nodes, peritoneal cavity and spleen of ID8 *pROSA*-iRFP720-bearing mice at week four post-inoculation and examined using a BD LSRFortessa X-20 (BD Biosciences). Percentage of (**A**) CXCR3+ CD4+ T cells, (**B**) CD8+ T cells, and (**C**) CCR4+ CD4+ T cells of the corresponding parent population. (**D**) Representative images of ovarian tumour sections stained with CD4 (yellow), CD8 (red) and CXCR3 (green) at four weeks post tumour inoculation. Nuclei were stained with DAPI (blue). Bar graphs show the percentage area of ovarian tumour sections stained with (i) CD4+CXCR3+ and (ii) CD8+CXCR3+. Images were acquired using the VS120 Virtual Slide Microscope (Olympus Corporation, Japan) and processed using the Olyvia software v2.9.1 (Olympus Corporation, Japan). Data were analysed by calculating the percentage area of CD4+CXCR3 or CD8+CXCR3+ colocalisation of total tissue area using a consistent binary threshold in ImageJ v1.0 (National Institute of Health, MD, USA). * = *p* < 0.05.

Consistent with its primary role in CD8+ Teff recruitment [37], we observed no change in CXCR3+CD4+T cells following sitagliptin treatment (Figure 5A). However, the percentage of CXCR3+CD8+ T cells in the peritoneal cavity was significantly increased, with a concomitant decrease in spleen (Figure 5B) suggesting directed trafficking between these compartments. This trend was also consistent with absolute CXCR3+CD8+ lymphocyte counts (Supplementary Figure S3B). There was no change in percentage of CCR4+CD4+ T cells (Figure 5C), suggesting that the increased lymphocyte trafficking induced by sitagliptin was CXCR3-dependent. Consistent with these changes, immunofluorescence staining confirmed the increased CD8+ and CXCR3+ T cell abundance and colocalisation in sitagliptin-treated tumour tissue; whilst there was no significant change in CD4/CXCR3 colocalisation (Figure 4D). This trend was also retained throughout disease progression (Supplementary Figure S3C), demonstrating a sustained shift in CXCR3+ mediated CD8+ lymphocyte migration and retention in both the peripheral and intratumoral microenvironment.

**Figure 6:**
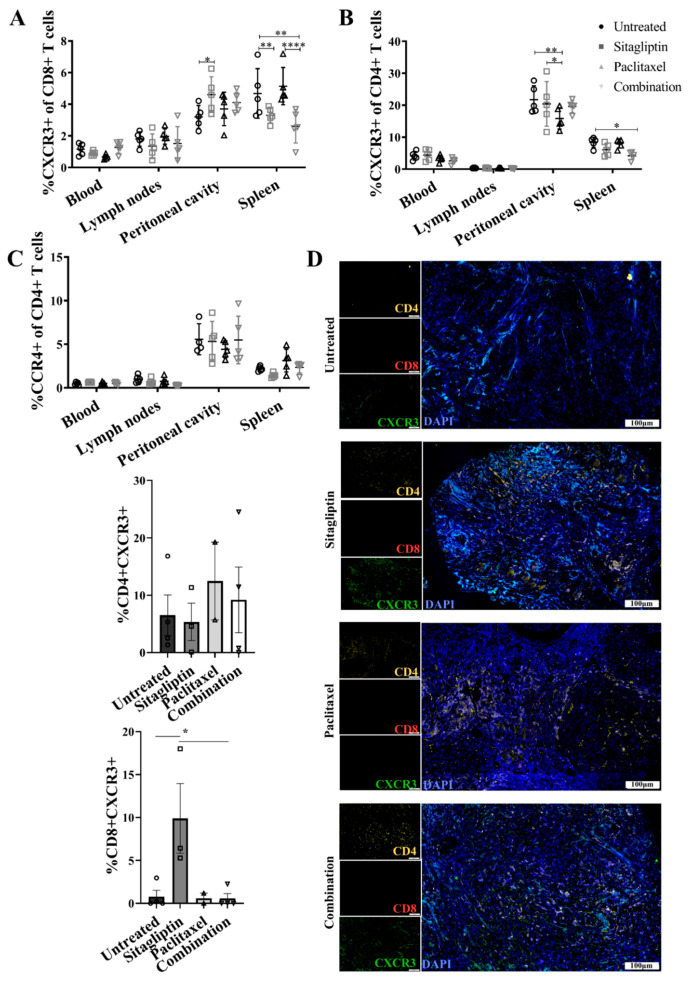
Chemokine receptor expression in ID8 tumour-bearing mice treated with sitagliptin and paclitaxel. Leukocytes were isolated from the blood, lymph nodes, peritoneal cavity and spleen of ID8 *pROSA*-iRFP720-bearing mice at week four post-inoculation and examined using a BD LSRFortessa X-20 (BD Biosciences). Percentage of (**A**) CXCR3+ CD8+ T cells, (**B**) CXCR3+ CD4+ T cells, and (**C**) CCR4+ CD4+ T cells of the corresponding parent population. (**D**) Representative images of ovarian tumour sections stained with CD4 (yellow), CD8 (red) and CXCR3 (green) at four weeks post tumour inoculation. Nuclei were stained with DAPI (blue). Bar graphs show the percentage area of ovarian tumour sections stained with (i) CD4+CXCR3+ and (ii) CD8+CXCR3. Images were acquired using the VS120 Virtual Slide Microscope (Olympus Corporation, Japan) and processed using the Olyvia software v2.9.1 (Olympus Corporation, Japan). Data were analysed by calculating the percentage area of CD4+CXCR3 or CD8+CXCR3+ colocalisation of total tissue area using a consistent binary threshold in ImageJ v1.0 (National Institute of Health, MD, USA). * = *p* < 0.05; ** = *p* < 0.01; **** = *p* < 0.0001.

To evaluate the relevance of sitagliptin-mediated lymphocyte trafficking to improve disease management in a clinical context, mice receiving sitagliptin were coadministered paclitaxel at one and two-weeks post-initiation of sitagliptin treatment. Paclitaxel was chosen as a representative second line therapy typically used for patients who have experienced disease relapse [38] when sitagliptin would likely be administered in a clinical context. Mice that received combination therapy exhibited decreased splenic percent CXCR3+CD8+ T cells (Figure 6A), as previously observed for sitagliptin alone. Unlike single therapy, however, the sitagliptin-paclitaxel combination did not induce a concomitant increase in peritoneal CXCR3+CD8+ T cells (Figure 6A). Furthermore, combination therapy also decreased percent CXCR3+CD4+ T cells in the spleen (Figure 6B). These results were recapitulated within the primary ovarian TME, where sitagliptin alone increased intra-tumoral CD8+/CXCR3+ expression and colocalisation but combination therapy did not (Figure 6D). Similar to sitagliptin-alone data, combination therapy did not alter CCR4-mediated migration (Figure 6C). Interestingly, paclitaxel-mediated inhibition of CXCR3+ T-cell recruitment to tumour tissue was temporary. Two weeks following final paclitaxel administration, the sitagliptin-mediated increase in CD8+/CXCR3+ expression and colocalisation in tumour tissue was restored (Supplementary Figure S3C). Our data demonstrate that daily administration of sitagliptin increased the recruitment and retention of CD8+ T cells into the tumour microenvironment. Furthermore, whilst active paclitaxel therapy temporarily inhibited CXCR3-mediated CD8+ T cell migration to tumour tissue, the effect was transient and could be reversed.

## 4. Discussion

The role of the immune system in the development and progression of ovarian cancer is well established [39], and overexpression of the enzyme DPP4 has been noted in several cancer types [1,11]) including ovarian cancer [13,40]. However, the role of DPP4 in various malignancies remains controversial. Recent work demonstrated that DPP4 inhibition can delay tumour progression in other malignancies [21,41]. However, the role of DPP4 and its potential as a therapeutic target in ovarian cancer has not been established. We provide the first evidence that sitagliptin can alter the tumour immune microenvironment in ovarian cancer in a prognostically desirable manner and may have utility as an adjunct to existing ovarian cancer therapies or emerging immunotherapies.

Our data demonstrate that sitagliptin delays tumour progression and extends survival of mice with ovarian tumours in vivo. The number and extent of metastases, particularly to the peritoneal wall, in mice receiving daily sitagliptin treatment was considerably reduced compared to controls. Similarly, previous studies have shown that DPP4 silencing or treatment with sitagliptin significantly reduced thyroid tumour growth in vivo [11]. Rats treated with sitagliptin had decreased precancerous lesions and reactive oxygen species in a colon cancer in vivo model [12], and DPP4 inhibition with sitagliptin suppressed tumour growth in a 4T1 syngeneic model of breast cancer [42]. Moreover, DPP4 inhibition with vildagliptin was also found to significantly reduce liver metastatic foci and metastatic index in a high fat diet-induced model of hepatocellular carcinoma [41]. It should also be considered that DPP4 is an adhesion molecule and can bind to fibronectin [43], which may influence metastasis. It is, therefore, essential to examine the effects of sitagliptin in an immune-deficient model to determine whether our observations are lymphocyte-dependent. It would also be interesting to examine epithelial-to-mesenchymal transition (EMT) in this context.

Surprisingly, whilst overall specific DPP4 activity decreased in serum, we observed that sitagliptin induced an overall increase in sDPP4 abundance, potentially due to increased sheddase expression subsequently releasing DPP4 from the cell surface [44]. Similar findings have been observed in HCC cell lines [45], and diabetic mice treated with the selective DPP4 inhibitor MK-0626 similarly exhibited a ~4-fold increase in circulating DPP4 [27]. Activated T-lymphocytes also display elevated secretion of sDPP4 and are a major source of sDPP4 in serum [46]. The relative contribution of DPP4 activity from each of these biological sources is unclear, however, with recent studies suggesting that circulating sDPP4 derived from multiple biological sources may contribute to different, nonoverlapping biological roles [47,48]. By contrast to serum, where only an ~50% decrease in specific activity was detected, we observed an almost complete abrogation of tumour-specific DPP4 abundance and activity following sitagliptin treatment. Similar tissue-specific effects have also been reported in liver and adipose tissue from animals treated with MK-0626 [27] Whilst the expression of DPP4 is ubiquitous and complex and can be influenced in a disease-specific manner [35,49], the mechanisms underlying tissue specific effects of sitagliptin remain unclear. However, our data do suggest that measurement of sDPP4 in serum is unlikely to provide a robust measurement to monitor the tumour-specific effects of sitagliptin in vivo.

We also identified increased expression of the CXCL10 receptor, CXCR3, on CD8+ T cells in the peritoneal cavity, and increased CXCR3+ expression in ovarian tumour sections following sitagliptin treatment. These data are consistent with previous work demonstrating preservation of full-length CXCL10 in the tumour microenvironment following DPP4 inhibition [21]. Furthermore, our group previously provided the first evidence for N-terminally cleaved antagonistic CXCL10 in malignant high-grade serous ovarian tumour samples but not benign ovarian disease and suggested that this was responsible for reduced lymphocyte infiltration in these tumours [35]. Whilst our study does not directly test a role for altered post-translational modification of CXCL10, the similarity in our results to the mechanisms described in da Silva et al. [21] strongly suggest the observed effects arise directly from CXCR3-mediated lymphocyte migration. By contrast to sitagliptin alone, the decreased splenic %CXCR3+CD8+ T cells in the combination group was not accompanied by increased peritoneal and ovarian TME CXCR3+CD8+ T cells. This may suggest that for mice receiving combination therapy, CXCR3+CD8+ T cells are migrating out of the spleen but not reaching their peripheral site. Paclitaxel targets tubulin and stabilises the microtubule polymer to prevent its disassembly, and microtubules play an essential role in directional cell migration initiated in response to chemokines [50]. Impairment of microtubule dynamics by paclitaxel may thus disrupt the migration of CXCR3+ T cells in response to the sitagliptin-mediated increased in bioactive CXCL10 [21], preventing their migration from spleen to their target site. However, the effect was reversible suggesting sitagliptin may provide clinical benefit through enhanced immune activation in patients not on active paclitaxel therapy. These results highlight the importance of timing optimisation when examining the efficacy of combined therapy in vivo and in clinical trials.

## 5. Conclusions

To summarise, we provided proof-of-concept evidence that the clinically approved drug sitagliptin can improve antitumour immunity in a syngeneic ovarian cancer mouse model, consequently reducing metastatic burden and prolonging survival. From a therapeutic perspective, our results provide a strategy to improve immune responses in ovarian cancer and establish a rationale for the use of DPP4 inhibitors as a rapidly translatable second-line treatment for this disease.

## Data Availability

The data presented in this study are available in this article and supplementary material.

## Supplementary Materials

The following are available online at https://www.mdpi.com/2072-6694/13/3/487/s1, Figure S1: Flow chart and timeline of study design, Figure S2: Flow cytometry gating strategy, Figure S3: MDSC percentages, CXCR3+ CD8+ lymphocyte absolute counts and intratumoral chemokine receptor expression in mice treated with sitagliptin +/− paclitaxel. Leukocytes were isolated from the blood, lymph nodes, peritoneal cavity and spleen of ID8 *pROSA*-iRFP720-bearing mice at week 4 post-inoculation.
