# Supplementary material for: DPP4 Inhibitor Sitagliptin Enhances Lymphocyte Recruitment and Prolongs Survival in a Syngeneic Ovarian Cancer Mouse Model
Source: Cancers (Basel). 2021 Jan 27;13(3):487. doi: 10.3390/cancers13030487 (PMC7865851; doi:10.3390/cancers13030487)
Supplement: Supplementary file 1 [file cancers-13-00487-s001.pdf]

cancers

MDPI

Supplementary materials: DPP4 Inhibitor Sitagliptin Enhances Lymphocyte Recruitment and Prolongs Survival in a Syngeneic Ovarian Cancer Mouse Model

Amy L. Wilson, Laura R. Moffitt, Kirsty L. Wilson, Maree Bilandzic, Mark D. Wright, Mark D. Gorrell, Martin K. Oehler, Magdalena Plebanski and Andrew N. Stephens

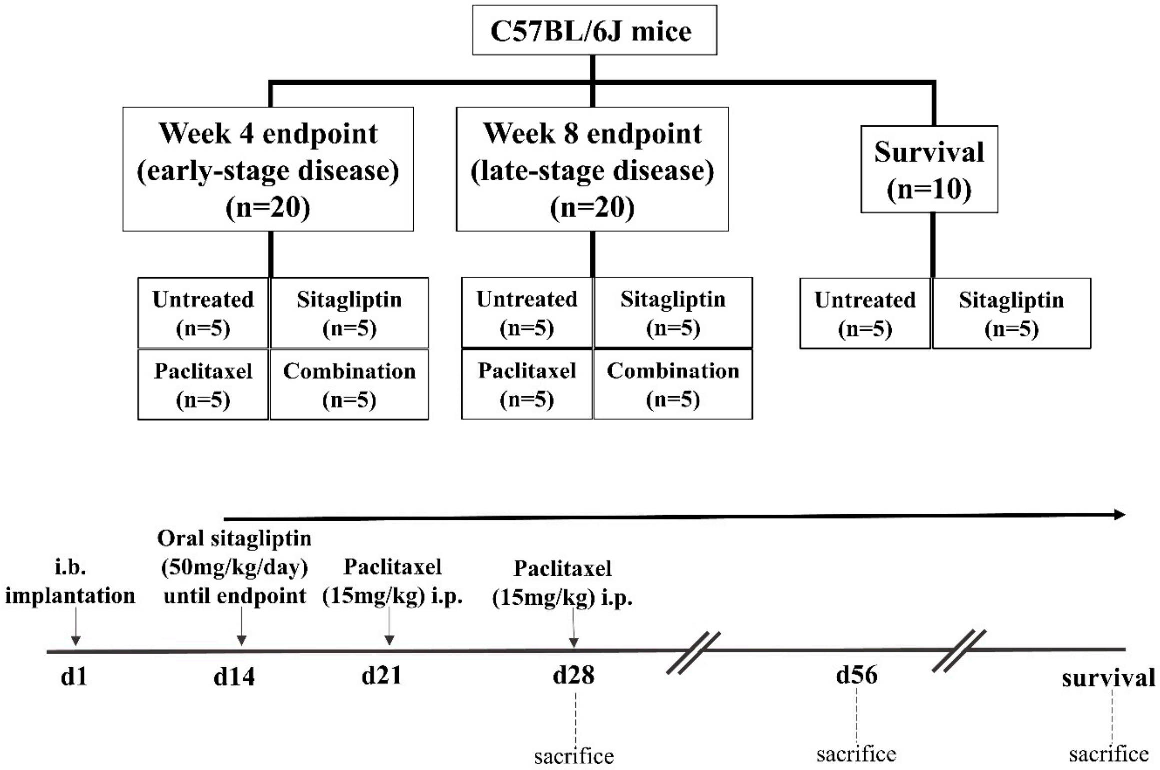

Figure S1. Flow chart and timeline of study design. Mice (n=5/group) were either untreated, or treated with sitagliptin, paclitaxel, or combination therapy and sacrificed at week 4 or week 8 post-experimental initiation, or were culled for survival analysis at humane endpoint. i.b.: intrabursal; i.p.: intraperitoneal.

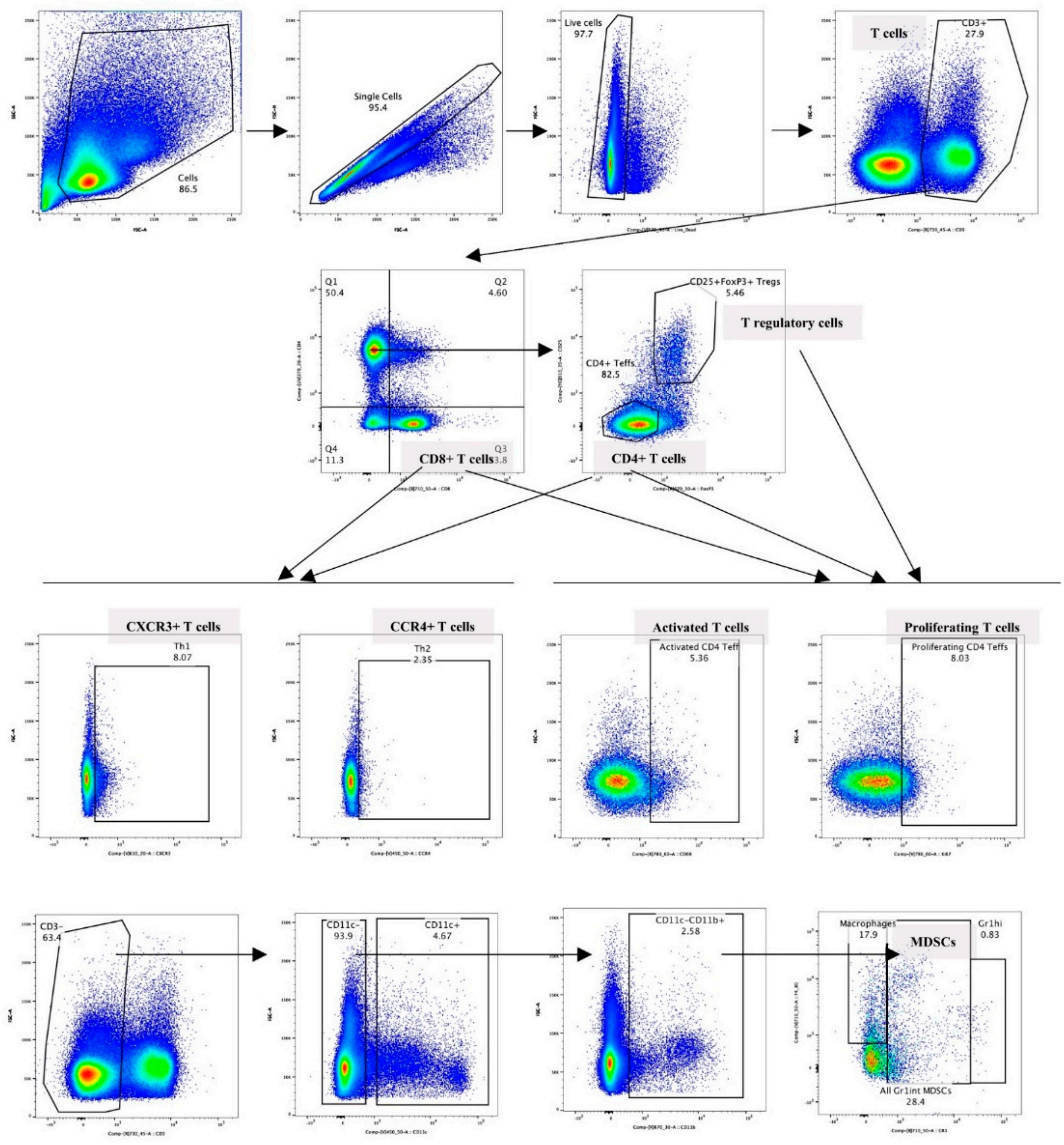

Figure S2. Flow cytometry gating strategy. All cells were defined on the FSC-A vs. SSC-A axis, and single cells were defined on the SSC-A vs. SSC-H axis. Dead cells were excluded using a LIVE/DEAD™ Fixable Aqua dead cell stain. T cells were defined as CD3+; CD8+ T cells were defined as CD3+CD8+; CD4+ T cells were defined as CD3+CD4+CD25-FoxP3-; T regulatory cells were defined as CD3+CD4+CD25+FoxP3+; migrating T cells were defined as CXCR3+ or CCR4+; activated cells were defined as CD69+ and proliferating cells were defined as Ki67+. Myeloid derived suppressor cells (MDSCs) were defined as CD3-CD11c-CD11b+Gr1int. All gates were set using fluorescence minus one (FMO) controls.

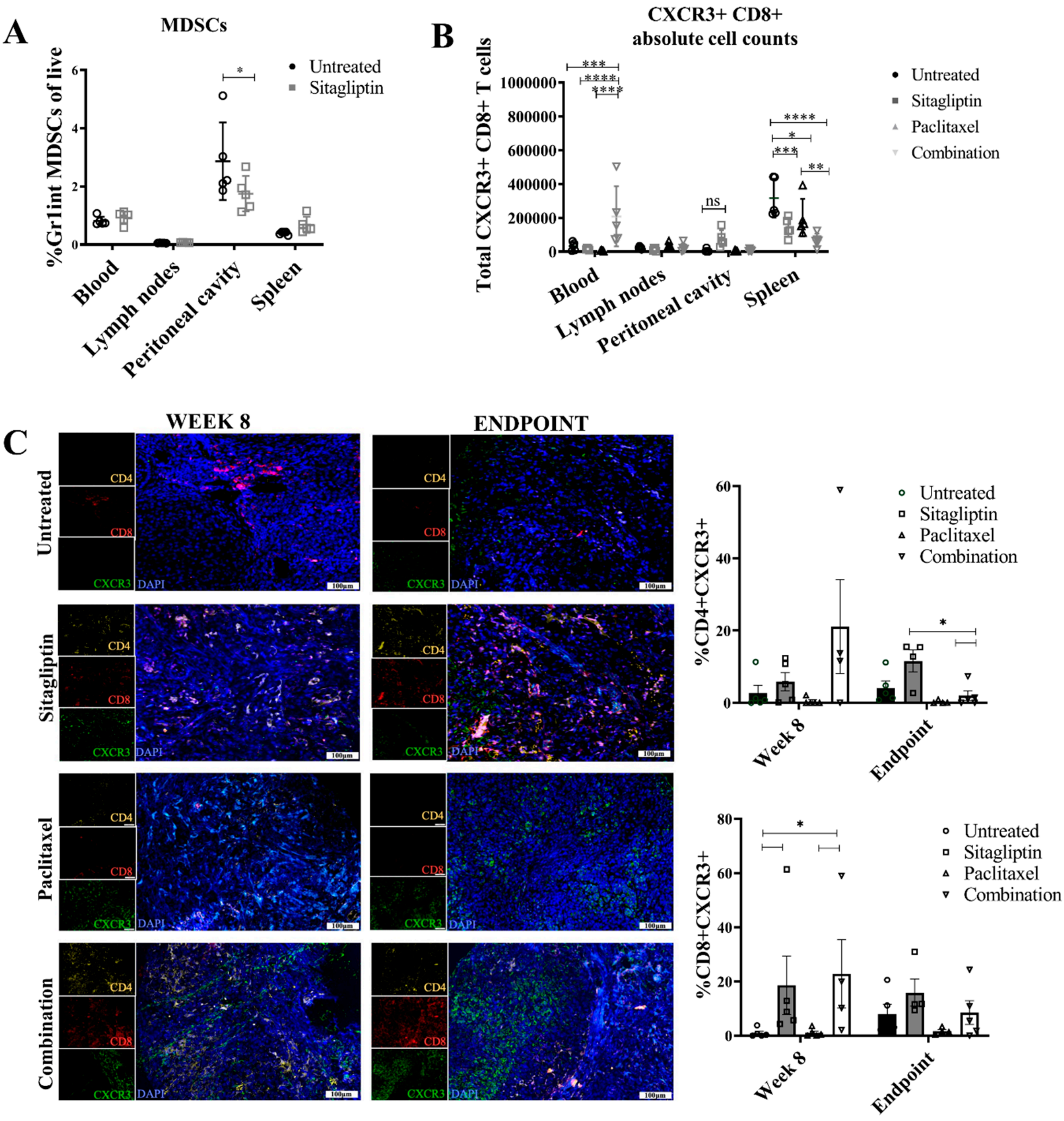

Figure S3. MDSC percentages, CXCR3+ CD8+ lymphocyte absolute counts and intratumoral chemokine receptor expression in mice treated with sitagliptin +/- paclitaxel. Leukocytes were isolated from the blood, lymph nodes, peritoneal cavity and spleen of ID8 *pROSA*-iRFP720-bearing mice at week 4 post-inoculation. (A) Percentage of myeloid-derived suppressor cells (MDSCs) (CD3-CD11c-CD11b+GR1int). (B) CD3+CD8+CXCR3+ absolute cell counts in each compartment. (C) Representative images of ovarian tumour sections stained with CD4 (yellow), CD8 (red) and CXCR3 (green) at 8 weeks post tumour inoculation and at endpoint. Nuclei are stained with DAPI (blue). Bar graphs show the percentage area of ovarian tumour sections stained with (i) CD4+CXCR3+ and (ii) CD8+CXCR3. Images were acquired using the VS120 Virtual Slide Microscope (Olympus Corporation, Japan) and processed using the Olyvia software v2.9.1 (Olympus Corporation, Japan). Data were analysed by calculating the percentage area of CD4+CXCR3 or CD8+CXCR3+ co-localisation of total tissue area using a consistent binary threshold in ImageJ v1.0 (National Institute of Health, MD, USA). Data are presented as mean ± SD, n=5. * = $p<0.05$; ** = $p<0.01$; **** = $p<0.0001$.
